# Manual and Spoken Cues in French Sign Language’s Lexical Access: Evidence From Mouthing in a Sign-Picture Priming Paradigm

**DOI:** 10.3389/fpsyg.2021.655168

**Published:** 2021-05-25

**Authors:** Caroline Bogliotti, Frederic Isel

**Affiliations:** ^1^UMR 7114, Modèles Dynamiques Corpus (MoDyCo), University Paris Nanterre, Nanterre, France; ^2^Institut Universitaire de France, Paris, France

**Keywords:** Lexical access, sign language, LSF, mouthing, sign-picture priming, intramodal bilingualism, bimodal bilingualism

## Abstract

Although Sign Languages are gestural languages, the fact remains that some linguistic information can also be conveyed by spoken components as mouthing. Mouthing usually tend to reproduce the more relevant phonetic part of the equivalent spoken word matching with the manual sign. Therefore, one crucial issue in sign language is to understand whether mouthing is part of the signs themselves or not, and to which extent it contributes to the construction of signs meaning. Another question is to know whether mouthing patterns constitute a phonological or a semantic cue in the lexical sign entry. This study aimed to investigate the role of mouthing on the processing of lexical signs in French Sign Language (LSF), according the type of bilingualism (intramodal vs. bimodal). For this purpose, a behavioral sign-picture lexical decision experiment was designed. Intramodal signers (native deaf adults) and Bimodal signers (fluent hearing adults) have to decide as fast as possible whether a picture matched with the sign seen just before. Five experimental conditions in which the pair sign-mouthing were congruent or incongruent were created. Our results showed a strong interference effect when the sign-mouthing matching was incongruent, reflected by higher error rates and lengthened reaction times compared with the congruent condition. This finding suggests that both groups of signers use the available lexical information contained in mouthing during accessing the sign meaning. In addition, deaf intramodal signers were strongly interfered than hearing bimodal signers. Taken together, our data indicate that mouthing is a determining factor in LSF lexical access, specifically in deaf signers.

## Introduction

### Spoken Components in a Signed Language

Reducing sign languages to their manual dimension is simplistic, as non-manual parameters are also used to produce messages. Several body parts – the whole face, gaze, eyebrows, chest, and mouth – play a linguistic role by bringing fine non-manual articulators to bear (Boyes-Braem et al., 2001; Woll, 2014; Johnston et al., 2016). The study of mouth actions is particularly relevant because it raises the issue of the influence of spoken and gestural languages contact on lexical access. To study the effect of mouthing on sign recognition, researchers must work at the interface of the linguistic, psycholinguistic, and sociolinguistic domains. To date, very few models of lexical access in sign language have been proposed and those few have focused on the role of sublexical elements such as location and handshape in relation to the neighborhood density effect during lexical access (see the spreading activation architecture proposed by Caselli and Cohen-Goldberg, 2014). The aim of this study was to understand what other sublexical factors in addition to location and handshape may play a determining role in the organization of and access to the mental lexicon in sign language. We particularly focused on the role of mouthing.

Our goal here was to search for behavioral evidence of the linguistic relevance of *mouthing* in accessing lexical information provided by signs in French Sign Language (LSF). Several studies (see below) have proposed that mouth actions can be divided into two types that are formally and functionally different: mouth gestures and mouthing. These two types of mouth actions are performed simultaneously with the manual sign and mobilize the mouth, lips, and tongue. One fundamental difference between the two categories of mouth actions is that while mouthing has a lexical function, mouth gestures convey more frequently morphosyntactic information. Crasborn et al. (2008) proposed a fine-grained typology of mouth actions to distinguish between mouth gestures and mouthing, based on three properties: (1) the independent or dependent meaning carried by the mouth; (2) whether the mouth action is or is not lexically associated with the manual sign; and (3) whether the mouth component is or is not borrowed from the ambient spoken language. In essence, a mouth gesture is frequently qualified as an oral component that is not derived from spoken language. More specifically, a mouth gesture can be an unvoiced syllable produced one or more times or an expiration of air, both of which echo the kinematic structure of the sign and are semantically empty (Woll, 2001; Crasborn et al., 2008). Woll and Sieratzki (1998) named this phenomenon *echo phonology*, with the idea that “the mouth ‘echoes’ the movement of the hand” (Johnston et al., 2016).

Other types of mouth gestures, which do have semantic content, exist: enaction (the mouth mimes the action meant by the sign, for example chewing gum); or adverbial/adjectival (the mouth gesture adds linguistic properties to the manual sign: a thin vs. large object or person represented by sunken cheeks vs. puffed cheeks, respectively). Mouth gestures are not borrowed from a spoken language and vary in the way that the manual component and mouth component are articulatorily and semantically associated.

Conversely, mouthing is a vocal production always borrowed from the surrounding spoken language, subvocalized or almost inaudible, and usually an approximation of the spoken word. Johnston et al. (2016) analyzed a large sample of AUSLAN (Australian Sign Language) corpora and highlighted different types of mouthing: the manual sign could be combined with a complete spoken word articulation or an incomplete articulation as the initial segment *diff(erent)*, the medial one *(re)mem(ber)*, the final segment *(im)prove*, or both initial and final segment *f(in)ish.* While mouthing is usually performed simultaneously with the manual sign, in some cases it may anticipate or follow the manual production.

Several studies based on video corpora analyses have investigated the frequency of mouthing, in different sign languages and among deaf signers (Boyes-Braem et al., 2001; Crasborn et al., 2008; Woll and Mesch, 2008). There is general agreement that mouthing, even though it is not systematic or obligatory, tends to co-occur with noun signs and fingerspelling, more rarely with verbs. However, in Japanese Sign Language, mouthing coexist with verbs (Penner, 2013). In addition to proposing the typology of mouth actions, Crasborn et al. (2008) compared the frequency of mouth actions in three typologically different sign languages (Dutch Sign Language, British Sign Language – BSL and Swedish Sign Language) and observed a similar tendency across all three sign languages: 50% to 80% of manual signs were produced with mouth actions, and mouthing was the most frequently occurring type of mouth action. This result suggests that mouthing is a useful clue to the lexical specification of a sign.

McKee (2007) reported that mouthing has various functions: (1) phonemic: mouthing can disambiguate two manual signs (e.g., in LSF, the signs meaning *chocolate* and *empty* are manually similar and are discriminated by mouthing); (2) morphemic: mouthing can specify or extend the meaning of a manual sign (e.g., in LSF, the mouthing of *apple* is articulated simultaneously with the manual sign EAT to produce the sentence “to eat an apple”; see several examples in Crasborn et al. (2008); (3) prosodic: to emphasize or stress the manual sign or bind elements within a clause (Weisenberg, 2003); (4) grammatical: to distinguish between nouns (mouthed) and verbs (not mouthed) (Kimmelman, 2009); and (5) psycholinguistic: to highlight the written/signed bilingual ability. Deaf people who use spoken language, in either the oral or written modality, tend to produce mouthing more frequently.

Although mouthing is a linguistic phenomenon observed in all sign languages studied, the question of whether it constitutes an inherent part of a lexical unit of sign languages has been raised. Johnston et al. (2016) suggested that, because mouthing is not obligatory, it is not part of the lexical representation of a sign and is more a code-blending phenomenon, i.e., a phenomenon observed in bimodal communication, characterized by the simultaneous production of signs and vocal words, than a spoken component of the lexical sign. Because there are no articulatory constraints, manual signs, and vocal speech can be produced simultaneously using different output channels (Emmorey et al., 2008a). Sociolinguistic and psycholinguistic factors can be invoked to explain more or less frequent use of mouthing, which is the trace of contact between the surrounding spoken language and the sign language. Johnston et al. (2016) suggested that English mouthing on AUSLAN signs is the consequence of contact of the second language (English) with the native one (AUSLAN) or may be related to oralist education (Bank et al., 2011). Boyes-Braem et al. (2001) reported that chronological age, age of acquisition and type of education (oralist vs. bilingual) could influence mouthing frequency, explaining that frequency of mouthing varies among individuals. Some researchers and deaf people themselves reported that more mouthing is produced when the communication occurs with a hearing speaker and this mouthing is louder. Some researchers strongly support the assumption that mouthing is not a real part of sign language and claim that it is just an optional complement of manual signs (Ebbinghaus and Heβmann, 2001). Ebbinghaus and Heβmann (2001) observed that the frequency of mouthing may dependent on exposure to the surrounding spoken language (Padden, 1980; Hohenberger and Happ, 2001), and suggested there is a relation between literacy and mouthing: the more literate the deaf signer is, the more frequent the mouthing will be.

All these studies investigated the linguistic characteristics of mouthing, but we also need to understand its psycholinguistic characteristics during processing. One fundamental question is how mouthing is processed by signers. What kind of information do signers use? How is mouthing represented in the lexicon of sign language? Muir et al. (2003) used an eye tracking technique with 8 deaf British Sign Language (BSL) signers while watching BSL video clips; they observed that the deaf participants’ gaze direction focused more on the characters’ faces than on their hands or body, which tended to mobilize peripheral vision. Most of the gaze points (75% to 90%) lay within 2.5° of the central regions (i.e., the face), with occasional rapid gazes toward other regions. Boutora and Meillon (2010) reported similar results with an eye-tracking pilot experiment with 3 LSF signers (2 deaf and 1 hearing) in which they have to understand a short story in LSF in order to resume it to the experimenter. The authors analyzed the eye gaze path between face and hands. Deaf and hearing signers did not use same gestural information when they perceived LSF utterances: while deaf signers focused mainly on the face, the hearing signer looked at both the face and the hands. To account for these different patterns of results, which were not discussed by the authors, we speculate that, since deaf signers are skillful at processing manual information, they have no problem simultaneously processing both manual and mouthing information. This may explain the results of Muir et al. (2003) and Boutora and Meillon (2010) indicating that deaf signers focus more on mouthing information and process manual information in the peripheral visual field. Furthermore, Huguet (2016) provided interesting results with an LSF eye-tracking study. She showed two LSF video clips to 4 deaf signers, one with the expected mouthing and one without mouthing. After each condition, participants gave a qualitative response to several questions, such as: Do you understand the video sentence? Does the absence of mouthing impede your understanding? The first result reported by Huguet was that the complete lack of mouthing strongly disturbed the deaf participants, who found it difficult to properly understand the meaning of the video. The second determining result in Huguet’s study concerned the eye movement data: the heat map revealed greater fixation point density on labial zone, even in the condition without mouthing in which no linguistic information was available there. This result suggests that the perceptual mechanism looks for crucial information in this part of the face. In addition, in the no mouthing condition, the author observed that the paths of eye movements were larger, and fixation points more spread out, undoubtedly because the deaf participants were searching for some “facial” linguistic information. Although these studies provide strong evidence that deaf signers use mouthing information in real time, little is known about how they use the mouthing information to access signs stored in the mental lexicon.

Regarding the role of mouthing in accessing the lexicon, Vinson et al. (2010) raised a central question about the semantic representations of mouthing. They ran an experimental study in BSL to investigate the extent to which mouthing and manual signs share semantic representations despite the fact that these two types of linguistic information do not use a common articulatory channel. They observed that mouthing and manual errors were dissociated, suggesting that they do not share same semantic representation. In addition, authors observed that mouthing’s semantic errors were more frequent in a picture-naming task than a word-translating task, suggesting that the presence of the orthography of the written word probably inhibited the semantic competition during lexical retrieval. The authors concluded that mouthing is not embedded in the manual component in the sign language lexicon. These results support the hypothesis that mouthing is not a “sign language phenomenon” and is not part of the sign language system. Addressing the same question, Giustolisi et al. (2017) ran a word–sign matching experiment in LIS (Italian Sign Language) to study the influence of mouthing on Italian word reading. They observed that deaf signers presented shorter reaction times in a condition in which there was strong mapping between mouthing and orthography. Since mouthing is highly facilitative, the authors argued that it is processed as phonemes and correlated with the spoken Italian lexicon, providing new evidence on the extrinsic status of mouthing in sign languages.

### Lexical Access in Spoken Language

The arbitrary nature of the relationship between the form and meaning of a word implies that it must be acquired and preserved, in one way or another, in the learner’s permanent memory. The expression “mental lexicon” is commonly used in psycholinguistics to refer to the body of knowledge that individuals have about words in their language (Segui, 2015). This knowledge concerns words’ semantic, syntactic, morphological, phonological and orthographic properties. Any model of language perception or production must necessarily include a lexical component. Indeed, the lexicon constitutes the fundamental interface that links the formal level to the interpretive level of language. If one accepts the principle that lexical knowledge is represented in the form of a mental dictionary (Treisman, 1960), the question arises of how one accesses the “entries” in this dictionary during word production and comprehension.

In cognitive psychology, almost all of the current models of language perception and production refer to the notion of activation. It is important to distinguish between activation and access. The activation of a lexical unit is a necessary but not sufficient condition for gaining access to the information that it contains. In addition to the notion of access to the lexicon, a crucial issue concerns the nature of the lexical representation in the mental lexicon. In the following section, we describe his point for sign languages. Several studies investigated the link between spoken and signed lexical access (Marshall et al., 2005; Kubus et al., 2015). One interesting result regarding sign/speech mental lexicon has been reported in Kubus et al.’s (2015) research. These authors studied visual word recognition in deaf bilinguals proficient in German Sign Language (DGS) and German. And investigated whether DGS signs are activated during a monolingual German word recognition task. They showed that lexical representations were associated cross-linguistically in the bilingual lexicon.

### Lexical Access in Sign Language

Although the study of lexical access in sign language is still in its infancy, the first study dates from the 1990s. Psycholinguistic studies of sign recognition aimed to determine whether the lexical recognition process is modality-specific or more general. In their review article on lexical access in sign language, Gutiérrez-Sigut and Baus (2021) reported a strong similarity between speech and sign processing. They showed that well-known lexical effects observed in spoken languages are also found in sign languages: lexicality, lexical frequency, and semantic priming. The seminal gating study by Emmorey and Corina (1990) investigated the role of manual sublexical information, that is, the three parameters of location, movement and handshape, in the sign recognition process. In their experiment, the gating task involved repeated presentation of a gestural sign, such that its duration from onset increased by a constant amount with each successive presentation. Their results highlighted the role of manual sublexical information in lexical access, and more specifically the singular role of each phonological parameter of a sign. They found that the location of the sign was identified first, followed by its manual handshape, and finally the movement made, which ensured the sign was recognized. Interestingly, these behavioral data were confirmed by a simulation conducted by Caselli and Cohen-Goldberg (2014) using a spreading activation architecture.

Although the same trends are observed in the lexical access process in sign language as in spoken language, the gestural modality influences the temporality of access. Because of the simultaneity of sublexical features and the minimal sequentiality, a sign is recognized faster than a spoken word: signs are recognized when around 35% of the sign has been produced, while words are recognized when around 80% of the word has presented (Emmorey and Corina, 1990; Gutiérrez-Sigut and Baus, 2021). These results are supported by the simulations generated by Caselli and Cohen-Goldberg’s (2014) computational model. Caselli and Cohen-Goldberg observed that model simulations matched the experimental data: the location parameter was activated earliest and seemed to be the most robust parameter due to its high sublexical frequency (the inventory of locations is smaller than those of handshapes or movements) and high perceptual saliency (location is the first parameter placed in the signing space, and due to its articulatory characteristics (i.e., more global motoric articulation), hold a large part of signing space. Consequently, this perceptual saliency led to a stronger memory encoding/trace that will improve sign production; Gutiérrez-Sigut and Baus, 2021).

Regarding to more modality-general effects, which are frequently observed in spoken languages, Baus et al. (2008) reported semantic interference in a picture-naming experiment in both native and non-native deaf signers of LSC (Catalan Sign Language). Deaf signers named pictures slower when they were presented in the semantically related condition than in the unrelated condition. The lexicality effect (sign or non-sign processing) has also been observed in several sign language studies (Emmorey, 1991; Emmorey and Corina, 1993; Carreiras et al., 2008; Guttiérez and Carreiras, 2009; Dye et al., 2016). In contrast, the lexical frequency effect is more difficult to investigate in sign language, because sign frequency databases are not yet available though they are being created for different sign languages (British Sign Language: Vinson et al., 2008; Fenlon et al., 2015; Australian Sign Language: Johnston, 2012; Spanish Sign Language: Gutiérrez-Sigut et al., 2016; American Sign Language: Caselli et al., 2017; French Sign Language: Perin et al., in progress). Sign language studies have found robust familiarity effects, which have been quantified with a Likert scale by deaf signers with a native or high level of proficiency in the respective sign language. In other words, familiar signs are recognized faster than less familiar signs (Spanish Sign Language: Carreiras et al., 2008 (American Sign Language: Emmorey and Corina, 1993; Mayberry and Witcher, 2005; Ferjan-Ramirez et al., 2016). Finally, some studies also noted a semantic priming effect in sign language using a sign-sign priming task, in both native and late signers (Emmorey and Corina, 1993; Mayberry and Witcher, 2005; Bosworth and Emmorey, 2010; for a review, see Gutiérrez-Sigut and Baus, 2021).

One question that remains open is which word/sign recognition characteristics are universal (language-general) in spoken and sign languages in models, and which are specific to each language modality (language-specific). To the best of our knowledge, few studies have investigated the role of mouthing in lexical access. In linguistic theory, some researchers consider mouthing to be part of the signs themselves, while others consider it to be an incidental consequence of language contact and not part of the sign lexicon (Sutton-Spence, 1999; Boyes-Braem et al., 2001; Sutton-Spence and Day, 2001; Nadolske and Rosenstock, 2007).

The question addressed in the present study concerns the status of mouthing, and more specifically the extent to which mouthing is a relevant cue to lexical processing in sign language, specifically LSF study. Should we consider mouthing as a sublexical feature or a semantic cue? Does the contact between spoken and sign information facilitate or inhibit the lexical access process? Does the spoken experience facilitate or inhibit the signed lexical access process? For this purpose, we designed a sign–picture priming experiment, in which participants had to decide if the manual sign–mouthing pair that composed the lexical sign fitted with the picture (Bishop, 2003; Friedrich and Friederici, 2008; Barcroft, 2009; Marinis, 2010). The congruency of the manual sign–mouthing pair varied. In our experimental design, the critical condition to assess the impact of mouthing on lexical decision was provided by interference between manual sign and mouthing. We predicted that condition would have an effect: incongruent conditions (semantic and phonological interference) should lead to higher error rates and longer decision times than congruent conditions. In addition, based on previous studies (see above), we hypothesized that the mouthing effects should vary according to the type of bilingualism: intramodal vs bimodal bilingualism. This should be reflected by more difficulties managing the manual–mouthing conflict of information in deaf signers than in hearing ones, since hearing signers could be less skilled at simultaneously processing manual and spoken information. Finally, we expect an interaction between condition and type of bilingualism with an increase of error rates and reaction times all the greater for the deaf Intramodal signers than the hearing bimodal ones.

## This Study

### Participants

Thirteen deaf native signers of LSF (*M _*age*_* 31;02 years; *SD* = 8;01 years), and 11 hearing native speakers of French who are fluent LSF signers (*M _*age*_* 27;02 years; *SD* = 8.;07 years) were recruited for this experiment (*M _*age*_* group difference *p* > 0.05). We called deaf signers *Intramodal bilinguals* given that they processed both sign and spoken language by the sole visual modality; LSF is their native or early language (exposure to LSF before 3 years of age). In contrast, hearing signers, either children of deaf adults (CODA, 2 participants) or French-LSF interpreters, are called in this study as *bimodal bilinguals* given that they processed sign and speech with two different channels (i.e., by the appropriate sensorial-perceptive modality, namely visuo-gestural and audio-oral). Except the two CODAs, for whom LSF was a native language, participants in this group learned LSF as a second language and they have a C2-Level (high skilled signers; Common European Framework of References for Languages). Their LSF/French interpreter function ensured robust sign language skills and frequent exposure to sign language, enough to imagine that mouthing could interfere with manual recognition. We collected metadata of each participant on their judgment for sign language proficiency using a self-rate Likert Scale from 1 (very low) to 6 (native). In average, Intramodal signers judged their sign language proficiency as 5 and Bimodal signers judged their sign language proficiency as 4.5. This result confirms that all participants have a high proficiency level in LSF. In addition, we calculated 1) the written French abilities on a Likert scale from 1 (low) to 3 (good), and 2) the spoken French exposure from 1 (rare) to 3 (frequent) for Intramodal signers. In average, the results showed that written French proficiency was assessed around 2.5 and for spoken French exposure 2.4. These results suggest that the Intramodal signers have a high level of familiarity with French.

### Stimuli

**FIGURE 1:**
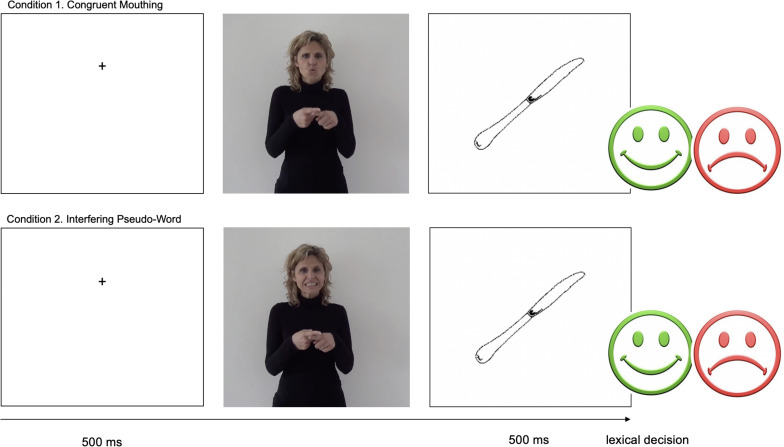
Flow chart for the lexical decision experiment. In the control condition, the congruent mouthing/kuto/feats with manual sign COUTEAU (/naɪf/KNIFE). In * Pseudo-Word condition, a pseudoword is presented with the manual sign KNIFE. In both conditions, participants have to decide if the picture matches the sign presented before it. All items are listed in the Supplementary Table 1.

There are no lexical norms for LSF signs. Consequently, we decided to use concrete signs belonging to current LSF lexicon, in the following semantic categories: fruits, vegetables, clothes, animals, objects and vehicles (see Supplementary Table 1 for complete list of stimuli). The stimuli were chosen according to the easiness of their pictorial representation. Isolated lexical signs (i.e., manual sign–mouthing pairs) were presented in five experimental conditions and participants had to decide if the lexical sign presented corresponded to the picture presented next on the screen. In the first condition *Control*, both sign and mouthing were congruent with the picture. In the second and third conditions, the sign was congruent with the picture but not with the mouthing: in the *Pseudo-Word*, the incongruent mouthing was a pseudoword; in the *Semantically incongruent*, the incongruent mouthing was a word semantically related to the sign. In the fourth condition, *Absence of Mouthing*, the sign was congruent with the picture and there was no mouthing. In the fifth condition, *Mouthing Alone*, the mouthing was congruent with the picture and there was no sign (Figure 1 and Table 1; all conditions are available in Supplementary Video 1). We created the stimuli in the incongruent mouthing conditions according to several factors: 1) the incongruent mouthing, either phonological either semantic, were fitted with the number of syllables of the congruent mouthing (e.g., the manual sign ARAIGNEE (SPIDER) was paired with a trisyllabic word, and respectively with the congruent mouthing/areɲe/in Control condition; with the pseudo-word/itufi/in Pseudo-Word condition, with/eskargo/in Semantically Incongruent condition; 2) the incongruent mouthing was created by paying careful attention to avoid a labial double, e.g., the pseudo-word/ʃifu/was visually too close of the word/ʃifʃ/and changed in/ʃifal/; 3) for Pseudo-Word condition, we payed attention to exaggerate the visual opposition between the expected congruent mouthing and the phonological mouthing (rounded lips, mouth aperture), e.g.,/ma/and/ti/syllables are strongly contrastive; and 4) for Semantically incongruent condition, the mouthing had to belong to the same semantic category, and to be close to the referent, e.g., the manual sign SPIDER can be paired with *snail* semantic incongruent mouthing, but not with *bear* or *dolphin.*

**TABLE 1:** The five experimental conditions presented in the lexical decision experiment.

Stimuli. MANUAL SIGN + /mouthing/	Picture in Related Condition (expected response: same)	Picture in Unrelated Condition (expected response: different)
Condition 1: Manual sign and congruent mouthing KNIFE + /naɪf/	KNIFE	CAT
*Condition 2: Manual sign and pseudo- word KNIFE + /p\~textopenofi/	KNIFE	SCOTTER
*Condition 3. Manual sign and semantically Incongruent mouthing KNIFE + /mɪksə^r^/	KNIFE	BELT
Condition 4: Manual sign and absence of mouthing KNIFE without mouthing	KNIFE	CAR
Condition 5: No manual sign and mouthing alone/naɪf/without manual sign	KNIFE	SPIDER

Interfering conditions are marked with an asterisk *. In the LSF experiment: Condition 1: COUTEAU + /kuto/; *Condition 2: COUTEAU+/p~pɔ̃fi/; *Condition 3: COUTEAU+/batœr/; Condition 4: COUTEAU without mouthing; Condition 5:/kuto/without manual sign.

For the unrelated pair (fillers), each manual sign-mouthing pair was associated to a picture that did not match with the sign (control condition). The pictures were taken from a standardized set of pictures (Snodgrass and Vanderwart, 1980).

### Procedure

As described in the section on Stimuli, we created five experimental conditions in which the combination of both the manual sign and the mouthing was systematically manipulated (Table 1). Participants had to make a lexical decision: they had to decide as fast as possible whether a picture matched the sign presented (Bishop, 2003; Friedrich and Friederici, 2008; Barcroft, 2009; Marinis, 2010). In each condition, 40 signs were presented.

The experiment was run using E-Prime 2 Software on a laptop computer. The screen background color was white. Each trial began with a black fixation cross (500 ms), followed by the sign video and then by the picture. The picture was displayed for 5000 ms. The intertrial interval was 3000 ms. The experiment was preceded by a short training block of 10 stimuli to familiarize participants with the experimental task. The instructions were given in LSF and were repeated by the researcher if some points were not clear to the participants. Given that the experiment aimed to study the processing of mouthing, we decided to induce participants to focus on the mouth. For this purpose, the fixation cross was located in the same place, as accurately as possible, where the mouth would appear on the screen with the stimuli. During the presentation of the picture, participants could press the green button (D key) if they thought the picture matched the lexical sign (*same*) or the red button (K key) if they thought the picture did not match the lexical sign (*different*). Participants had a maximum of 5000 ms to respond (Figure 1). Participants were given 400 stimuli, which were distributed in 10 blocks of 40 stimuli each. In each block, 8 stimuli in each of the 5 conditions were presented, with half of the stimuli presented in the related condition and the other half in the unrelated condition (fillers). Block order was counterbalanced across participants and stimuli were pseudo-randomized within each block.

### Predictions

For Bilingualism, we expect both higher Error Rates and longer Reaction Times (RT) for Bimodal than Intramodal due to possible interference between gestural and spoken linguistic channel in hearing bimodal signers.

For Condition, the following pattern for both ER and RT should be observed: *Control* < *Absence of mouthing* < *Pseudo-Word* < *Semantically incongruent* < *Mouthing Alone*.

At a more fine-grained level, the five hypotheses were formulated for both ER and RT:

- H1. Higher Error Rates and longer Reaction Times for Pseudo-Word than Control.
- H2. Higher Error Rates and longer Reaction Times for Semantically Incongruent than Control.
- H3a. If mouthing plays a role in lexical access, then Error Rates in the Absence of Mouthing condition should be higher, and ReactionTimes longer, than in the Control one.
- H3b. Else no Error Rates differences between Absence of Mouthing and Control should be observed.
- H4. Error Rates should be higher, and Reaction Times longer, in Mouthing Alone than in Control.
- H5a. Under the hypothesis that during sign processing there is retrieval of the lexical information conveyed by mouthing, then the Semantically Incongruent condition should give rise to higher Error Rates, and longer Reaction Times, than the Pseudo-Word conditions (which contains no lexical information).
- H5b. Else, no difference should be found between Semantically Incongruent and Pseudo-Word conditions.

Finally, for Error rates, we expect a Bilingualism by Condition interaction reflecting higher error rates for bimodal signers in condition favoring a possible gestural and spoken interference (Pseudo-Word & Semantically incongruent).

For Reaction Times, a Bilingualism by Condition interaction should also be observed. Whereas longer Reaction Times for Bimodal compared to Intramodal bilinguals should be found in the comparisons implying both gestural and spoken channels (H1, H2), longer Reaction Times should be expected for Intramodal in comparison with Bimodal for Mouthing alone (H4).

## Results

We ran two analyses of variance (ANOVAs), one on error rates and one on reaction times. Incorrect responses were excluded for the reaction time analysis. ANOVAs were run with Bilingualism (2 levels: intramodal vs. bimodal) as between-subject factor, and Condition (5 levels: Control, Pseudo-Word, Semantically incongruent, Absence of mouthing, Mouthing alone) as within-subject factor. Before running the statistical analyses, we first computed the interval [mean ± 2SD]. Results that were outside of this interval (8.5% on average for Error Rate and 2.5% on average for Reaction Time) were considered as outliners and therefore were excluded of our statistical analysis.

### Error Rates

**FIGURE 2:**
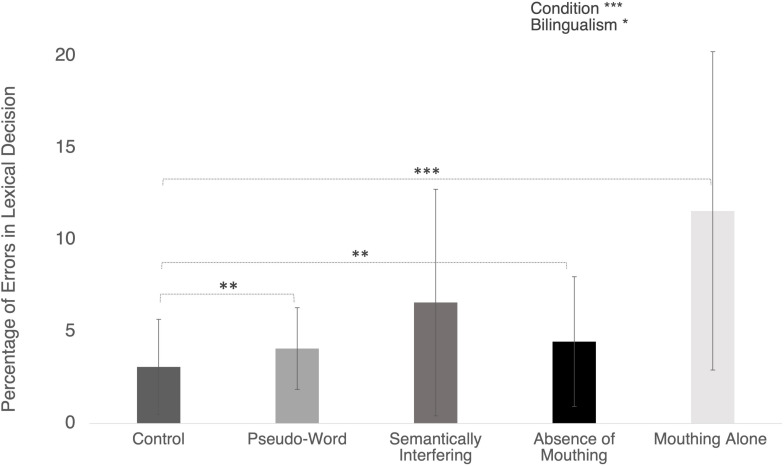
Error rates (in percentage) in the different conditions. Error bars represent standard deviations. Significant post hoc comparisons are indicated with conventional asterisks (^∗^*p* < 0.05; ^∗∗^*p* < 0.01; ^∗∗∗^*p* < 0.001).

The ANOVA revealed that the main effect of Bilingualism was significant [*F*(1,16) = 5.51; *p* = 0.032; η_p_^2^ = 25.61%; Figure 2), indicating that on average Intramodal made fewer errors than Bimodal signers (respectively *M* = 3.7%, *SD* = 2.9% and *M* = 6.9%, *SD* = 4.9%).

The main effect of Condition also reached the significance level [*F*(4,64) = 6.25; *p* < 0.001; η_p_^2^ = 28.08%; see Table 2). Further *post hoc* tests using the Bonferroni corrected threshold of *p* < 0.001 showed that the mean error rate was significantly higher in 1) Pseudo-Word condition (*M* = 4.1%, *SD* = 2.2%, *p* = 0.004), 2) Absence of Mouthing (*M* = 3.7%, *SD* = 2.9%, *p* = 0.002), and 3) Mouthing Alone (*M* = 9.2%, *SD* = 5.7%; *p* = *0.001*) than in Control condition (*M* = 2.9%, *SD* = 2.4%).

**TABLE 2:** Error rates (in percent) and Reaction Times (in ms) in the five conditions according to bilingualism (Intramodal vs. Bimodal).

	Control	Pseudo-word	Semantically incongruent	Absence of mouthing	Mouthing alone
**Intramodal signers**					
Error rates (SD)	2.8 (1.6)	3.4 (1.7)	5.9 (6.2)	2.3 (2.3)	5.0 (3.1)
Reaction times (SD)	708.9 (231.4)	824.8 (324.9)	890.2 (410.5)	743.2 (247.7)	801.9 (319.0)
**Bimodal signers**					
Error rates (SD)	4.0 (3.6)	4.8 (2.8)	7.3 (6.2)	5.0 (3.5)	13.3 (8.3)
Reaction times (SD)	535.8 (163.6)	574.9 (198.6)	590.1 (205.2)	586.1 (201.6)	722.4 (312.1)

Finally, the ANOVA failed to show a significant Bilingualism by Condition interaction (F < 1).

### Reaction Times

**FIGURE 3:**
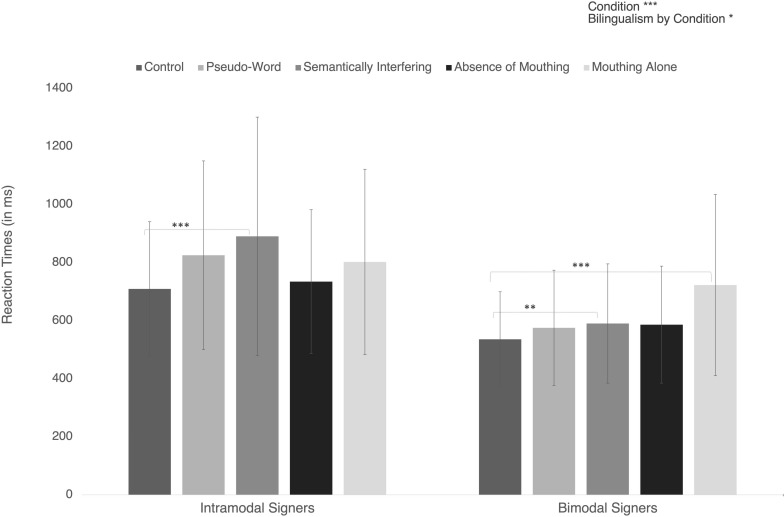
Reaction times in the different in the two groups of bilinguals. Error bars represent standard deviations. Significant post hoc comparisons are indicated with conventional asterisks (^∗^*p* < 0.05; ^∗∗^*p* < 0.01; ^∗∗∗^*p* < 0.001).

The ANOVA only showed a significant main effect of Condition [*F*(4,76) = 8.06; *p* < 0.001; η_p_^2^ = 29.78%), indicating that reaction times varied as a function of the different experimental conditions (see Table 2 and Figure 3).

Further *post hoc* analyses with a Bonferroni corrected threshold at *p* < 0.005 indicated that mean reaction times were significantly shorter in Control than in Pseudo-Word condition (respectively *M* = 629.6 ms, *SD* = 217.6 ms, and *M* = 716.2 ms, *SD* = 299.6 ms; *p* = 0.004) or in Semantically incongruent one (*M* = 759.7 ms, *SD* = 363.7 ms; *p* < 0.001).

Finally, Mouthing Alone significantly lengthened the mean reaction time (*M* = 765.7 ms, *SD* = 310.9 ms) in comparison with Control condition (*M* = 629.6 ms, *SD* = 217.6 ms; *p* = 0.003).

There was also a significant Bilingualism by Condition interaction [*F*(4,76) = 2.50; *p* = 0.049; η_p_^2^ = 11.64%). This interaction indicates that the difference between Reaction Times in Semantically incongruent condition and Control conditions was greater in Intramodal signers (respectively *M* = 759.7 ms vs. *M* = 629.6 ms; *d* = 181.3 ms, *p* = 0.001) than in Bimodal signers (respectively *M* = 590.1 ms vs. *M* = 535.7 ms; *d* = 54.3 ms, *p* = 0.003). In addition, reaction times were significantly longer in Mouthing Alone condition than in Control one for Bimodal signers (*M* = 722.4 ms vs. *M* = 535.8 ms; *p* < 0.001) but not for Intramodal ones (*p* > 0.05).

## Discussion

As Schermer (1985) claimed, “the existence of a pure sign language, without the occurrence of any speech, among deaf adults, is more or less a theoretical construct” (p. 288; cited in Bank et al., 2015). We know now that sign language is not solely a manual language, and it must be recognized that mouthing may play a role in sign recognition. In this study, we aimed to investigate the “spoken cue” in sign language, by specifically investigating the role of mouthing in the lexical access. To do this, we decided to create an experimental task to compare error rates and reaction times in different conditions in which mouthing matched or did not match the produced manual signs. We know that age and frequency of exposure to a sign language have a strong impact on sign recognition, but the more relevant issue here was analyzing the effect of Intramodal exposure (only sign language) vs. bimodal exposure (spoken language in addition to a gestural one). More specifically, what is interesting here is the fact that deaf signers perceive spoken cues through the visual modality (“seen speech”; Capek et al., 2008). As reported in the literature on reading by the deaf, deaf signers develop some phonological awareness and may activate a kind of silent phonology (MacSweeney et al., 2008; Hirshorn et al., 2015). So, we can postulate that LSF signers have phonological representations, undoubtedly incomplete, of French spoken words. From these observations, we hypothesized that signers used the spoken stream to retrieve lexical information. To evaluate this modality effect, we administered our experimental task to two groups: (1) a group of deaf native signers of LSF, that is, Intramodal bilinguals, with no access to any spoken language in its audio-aural modality; and (2) hearing signers whose native language was French, that is, bimodal bilinguals, with a high proficiency in LSF because they work as LSF–French interpreters.

This study revealed that signers exploit mouthing to recognize signs and to retrieve linguistic information in the signed lexicon: accuracy was lower in Pseudo-Word condition than in Control one, and Reaction Time was lengthened in both Pseudo-Word and Semantically incongruent conditions than in the Control condition. More precisely for Reaction Times, whereas the effect for Semantically Incongruent was 117.8 ms, it was only 77.5 ms for Pseudo-Word. Interestingly, we showed a trade-off effect between accuracy and speed for incongruent conditions: Intramodal Signers were more accurate but slower to make a sign lexical decision compared to the Bimodal Signers. In contrast, Bimodal signers answered faster but did more errors in comparison with Intramodal Signers. This result suggests that Bimodal and Intramodal signers used different the processing strategies. Taken together, these findings suggest that mouthing may play a determining role at a lexical-semantic stage of processing.

As mentioned above, we aimed to assess the role of mouthing in lexical access as a function of bilingualism, the Intramodal signers (deaf native signers) vs. Bimodal ones (hearing fluent signers). In our study, during sign language processing, Intramodal signers seem to be functionally bimodal and Bimodal signers functionally Intramodal: Intramodal signers use both spoken and manual input, while bimodal signers seem to prefer manual information and ignore mouthing. One surprising result concerned the number of errors made by Bimodal signers: they produced more errors than Intramodal signers in Mouthing Alone condition, in which the lexical decision was made from the mouthing cue alone (Bimodal: 13.3% *SD* = *8.3;* Intramodal: 5.0% *SD* = *3.1*). In addition, Bimodal signers presented consistent response times in all experimental conditions, except in the Mouthing Alone condition in which reaction times were strongly lengthened (around 150 ms slower than the 4 other conditions with the manual sign). These results suggest that bimodal signers paid less attention to the mouthing whatever the experimental condition. We hypothesized that the “spoken condition” would be successfully processed by the “spoken participants.” But contrary to our expectations, Intramodal signers performed better than Bimodal signers at processing mouthing. One possible explanation is that the Bimodal signers’ processing strategy was to rely on manual signs when making lexical decisions because they are more salient and more robust, and that mouthing was too interferent for them. We hypothesize that mouthing is not a sufficiently reliable cue for them to process the sign, and they strategically decide to make their lexical decisions by focusing on manual information. These latter could adopt a strategy to process the lexical unit by choosing the more reliable sensorial modality to process it, focusing on spoken trace or manual cue. Previous results of Emmorey et al. (2008b) suggested that hearing signers used frequently mouthing to process signs. But, contrary to our study, these signers were beginning signers and they used the spoken cue to support sign processing when they need any helpful semantic information. In our study, hearing signers were highly fluent signers, and this may have as a consequence to adopt the strategy to focus only on manual sign and consciously ignore the mouthing in order to not be interfered in lexical decision.

Regarding to Intramodal signers, we observed another type of lexical processing. These results seem give new evidence about the semantic role of mouthing in the lexical retrieval and question us about its possible involvement in the semantic representation. Intramodal signers’ better performance in our study reinforces results previously reported by Huguet (2016) and Muir et al. (2003), in which deaf signers experienced difficulties when they had to process signs without mouthing. Intramodal signers focused on the face and lips, suggesting that this zone provides relevant information during lexical access. Taken together, these studies confirm that Intramodal signers use mouthing information to assist with lexical access if it is available. Several studies on sign languages argue that mouthing is optional and useful only to disambiguate the meanings of two signs and claim that is proof that mouthing is not a component of sign languages. We disagree with this claim as (1) Reaction Times were significantly lengthened when mouthing does not match with signs as in Pseudo-Word and Semantically Incongruent condition, and (2) accuracy was lower in Pseudo-Word condition compared to Control condition. This finding provides new evidence that mouthing supports manual sign processing in signers, particularly deaf signers and highlight the specificity of processing of spoken cues by deaf and hearing signers.

Although we failed to show an effect of presence of congruent mouthing on sign recognition, however it is important to note that an incongruent mouthing produce more errors and/or longer reaction times. In addition, previous eye-tracking studies demonstrated that the deaf signers rely more on mouth information than manual one in comparison with either hearing or late signers. We do not support the conclusion of Vinson et al. (2010) and Giustolisi et al. (2017) who claimed that mouthing is external to the lexical representations of signs. As we described previously, mouthing is not mandatory, but it seems be a reliable cue to facilitate lexical access. If our results cannot clearly support that mouthing made part of lexical representation of a sign, we wonder the reason for these authors supporting an external role of mouthing in lexical access. Maybe we can consider mouthing as a linguistic component that is acquired later because it is less salient than manual cues. Or mouthing may be a later stage of language development because it is related to the spoken language or has a spoken origin. During the developmental trajectory, children focused and used different cues, according to the integrity and maturity of their perceptual and processing systems. As soon as they learned the writing system of spoken language, their lexical processing should be influenced by this knowledge. Then the surrounding spoken language would be mastered, and the spoken cue (mouthing) could be used to process a sign and integrated to the specification of the sign. So, further investigations are necessary to provide evidence about the semantic role of mouthing in lexical access.

In addition, Giustolisi et al. (2017) suggested that a complete sign requires the specification of all manual parameters, while mouthing can be dropped from this specification. We disagree with this view because missing or impaired information about one of the manual parameters can have the same consequence as missing or impaired mouthing: lexical retrieval is inaccurate or slowed. This is not sufficient evidence neither to conclude that mouthing is part of sign language, nor to conclude that is not part of.

Finally, we propose that researchers are inaccurate in describing mouthing as a trace of spoken language. In Capek et al.’s (2008) article, they refer to *seen speech* to talk about mouthing. We believe it is essential to adopt this terminology to conduct a fine-grained analysis of mouthing. At first glance, mouthing is a spoken component from the surrounding spoken language attached to a sign language. But mouthing is more than just a word from spoken language: it is a loan that has been phonologically adapted. Deaf people cannot acquire complete phonological information, so spoken information is reduced to a gesture, a seen speech gesture. To study the relation between lipreading and phonological representation in deaf people, one way could be to use a mediated priming paradigm, varying indirectly the lipreading prime and the target picture (i.e., lipreading prime *car* - reduced form of carpet-, and a target picture of ship).

Future behavioral and neurophysiological studies are needed to test the role of mouthing during lexical access in sign language in different populations of signers. In particular, a hybrid dual-route architecture taking mouthing into consideration may be relevant to account for sign recognition in sign language. As our study suggests that Intramodal and Bimodal signers may not rely on mouthing in the same way to access the lexicon, we propose a first version of a processing model for LSF signs. This speculative model postulates that according to the type of signers (i.e., intramodal or bimodal) different processing strategies might be used. These strategies could be captured in a functional architecture postulating two processing routes to access the lexicon in sign language. A first route, the direct route, would constitute a direct mapping between the parameters of signs (manual such as location, handshape and movement) contained at a sublexical level and the stored lexical representation of each sign. This direct route is preferentially used by Bimodal signers. The direct route depends on processing a holistic representation of the sign. A second route, the decompositional route, an analytic one, involved the mouthing processing during sign lexical access. This latter route may be preferentially used by intramodal signs which need to rely on the analysis of the different parameters constituting each sign, including mouthing. Taken together, the data of our study suggest that mouthing information supports the processing system in order to facilitate recognition of signs that need to be assisted by mouthing.

## Data Availability Statement

The original contributions presented in the study are included in the article/Supplementary Material, further inquiries can be directed to the corresponding author.

## Ethics Statement

The studies involving human participants were reviewed and approved by the Local Ethics Committee of the Paris Nanterre University – Department of psychology and was performed in accordance with the Declaration of Helsinki. The participants provided their written informed consent to participate in this study.

## Author Contributions

CB: conceptualization, methodology, investigation, and writing – original draft preparation. Funding Acquisition FI: conceptualization, methodology, writing – original draft preparation. All authors contributed to the article and approved the submitted version.

## Conflict of Interest

The authors declare that the research was conducted in the absence of any commercial or financial relationships that could be construed as a potential conflict of interest.

## Supplementary Material

Supplementary Table 1 List of stimulus items. The table lists all 400 items presented in the lexical decision experiment.

Supplementary Video 1 Pomme (Apple) manual sign presented in the five conditions.

## References

[B1] Bank R. Crasborn O. van Hout R. (2015). Alignment of two languages: The spreading of mouthings in Sign Language of the Netherlands. *International Journal of Bilingualism* 19 40–55. 10.1177/1367006913484991

[B2] Bank R. Crasborn O. A. van Hout R. (2011). Variation in mouth actions with manual signs in Sign Language of the Netherlands (NGT). *Sign Language & Linguistics* 14 248–270. 10.1075/sll.14.2.02ban 33486653

[B3] Barcroft J. (2009). Effects of Synonym Generation on Incidental and Intentional L2 Vocabulary Learning During Reading. *TESOL Quarterly* 43 79–103. 10.1002/j.1545-7249.2009.tb00228.x

[B4] Baus C. Gutiérrez-Sigut E. Quer J. Carreiras M. (2008). Lexical access in Catalan Signed Language (LSC) production. *Cognition* 108 856–865. 10.1016/j.cognition.2008.05.012 18656181

[B5] Bishop D. V. (2003). *Test for reception of grammar* (Harcourt Assessment). London: Psychological Corporation.

[B6] Bosworth R. G. Emmorey K. (2010). Effects of iconicity and semantic relatedness on lexical access in american sign language. *Journal of Experimental Psychology: Learning, Memory, and Cognition* 36 1573–1581. 10.1037/a0020934 20919784 PMC2970771

[B7] Boutora L. Meillon B. (2010). *Utilisation d’outils occulométriques dans le repérage d’indices visuels de la LSF en perception*. Montreal: Atelier TALS.

[B8] Boyes-Braem P. Sutton-Spence R. Rijksuniversiteit te Leiden. (2001). *The hands are the head of the mouth: The mouth as articulator in sign languages*. Hamburg: Signum Press.

[B9] Capek C. M. Waters D. Woll B. MacSweeney M. Brammer M. J. McGuire P. K. (2008). Hand and Mouth: Cortical Correlates of Lexical Processing in British Sign Language and Speechreading English. *Journal of Cognitive Neuroscience* 20 1220–1234. 10.1162/jocn.2008.20084 18284353 PMC3370423

[B10] Carreiras M. Gutiérrez-Sigut E. Baquero S. Corina D. (2008). Lexical processing in Spanish Sign Language (LSE). *Journal of Memory and Language* 58 100–122. 10.1016/j.jml.2007.05.004

[B11] Caselli N. K. Cohen-Goldberg A. (2014). Lexical access in sign language: A computational model. *Frontiers in Psychology* 5:428. 10.3389/fpsyg.2014.00428 24860539 PMC4030144

[B12] Caselli N. K. Sehyr Z. S. Cohen-Goldberg A. Emmorey K. (2017). ASL-LEX: A lexical database of American Sign Language. *Behavior Research Methods* 49 784–801. 10.3758/s13428-016-0742-0 27193158 PMC5116008

[B13] Crasborn O. A. van der Kooij E. Waters D. Woll B. Mesch J. (2008). Frequency distribution and spreading behavior of different types of mouth actions in three sign languages. *Sign Language & Linguistics* 11 45–67. 10.1075/sll.11.1.04cra 33486653

[B14] Dye M. Seymour J. Hauser P. (2016). Response bias reveals enhanced attention to inferior visuel field in signers of American Sign Language. *Experimental Brain Research* 4 1067–1076. 10.1007/s00221-015-4530-3 26708522

[B15] Ebbinghaus H. Heβmann J. (2001). “Sign language as multidimensional communication: Why manual signs, mouthings, and mouth gestures are three different things,” in *The hands are the head of the mouth. The mouth as articulator in sign languages*, eds Boyes-Braem P. Sutton-Spence R. (Hamburg: Signum Press), 133–151.

[B16] Emmorey K. (1991). Repetition priming with aspect and agreement morphology in American Sign Language. *Journal of Psycholinguistic Research* 20 365–388. 10.1007/BF01067970 1886075

[B17] Emmorey K. Borinstein H. B. Thompson R. Gollan T. H. (2008a). Bimodal bilingualism. *Bilingualism: Language and Cognition* 11 43–61. 10.1017/S1366728907003203 19079743 PMC2600850

[B18] Emmorey K. Thompson R. Colvin R. (2008b). Eye Gaze During Comprehension of American Sign Language by Native and Beginning Signers. *Journal of Deaf Studies and Deaf Education* 14 237–243. 10.1093/deafed/enn037 18832075

[B19] Emmorey K. Corina D. (1990). Lexical Recognition in Sign Language: Effects of Phonetic Structure and Morphology. *Perceptual and Motor Skills* 71(3_suppl) 1227–1252. 10.2466/pms.1990.71.3f.1227 2087376

[B20] Emmorey K. Corina D. (1993). Hemispheric specialization for ASL signs and english words: Differences between imageable and abstract forms. *Neuropsychologia* 31 645–653. 10.1016/0028-3932(93)90136-N 8371838

[B21] Fenlon J. Cormier K. Schembri A. (2015). Building BSL SignBank: The Lemma Dilemma Revisited. *International Journal of Lexicography* 28 169–206. 10.1093/ijl/ecv008

[B22] Ferjan-Ramirez N. Leonard M. K. Davenport T. S. Torres C. Halgren E. Mayberry R. I. (2016). Neural Language Processing in Adolescent First-Language Learners: Longitudinal Case Studies in American Sign Language. *Cerebral Cortex* 26 1015–1026. 10.1093/cercor/bhu273 25410427 PMC4737603

[B23] Friedrich M. Friederici A. D. (2008). Neurophysiological correlates of online word learning in 14-month-old infants. *NeuroReport* 19(18) 1757–1761. 10.1097/WNR.0b013e328318f014 18955904

[B24] Giustolisi B. Mereghetti E. Cecchetto C. (2017). Phonological blending or code mixing? Why mouthing is not a core component of sign language grammar. *Natural Language & Linguistic Theory* 35 347–365. 10.1007/s11049-016-9353-9

[B25] Gutiérrez-Sigut E. Baus C. (2021). “Lexical processing in sign language comprehension and production,” in *The Routledge Handbook of Theorical and Experimental Sign Language Research*, eds Quer J. Pfau R. Herrmann A. (London: Routledge), 10.31219/osf.io/qr769

[B26] Gutiérrez-Sigut E. Costello B. Baus C. Carreiras M. (2016). LSE-Sign: A lexical database for Spanish Sign Language. *Behavior Research Methods* 48 123–137. 10.3758/s13428-014-0560-1 25630312

[B27] Guttiérez E. Carreiras M. (2009). *El papel de los parámetros fonológicos en el procesamiento de los signos de la*. LSE. Madrid: Fundación CNSE.

[B28] Hirshorn E. A. Dye M. W. G. Hauser P. Supalla T. R. Bavelier D. (2015). The contribution of phonological knowledge, memory, and language background to reading comprehension in deaf populations. *Frontiers in Psychology* 6:1153. 10.3389/fpsyg.2015.01153 26379566 PMC4548088

[B29] Hohenberger A. Happ D. (2001). “The linguistic primacy of signs and mouth gestures over mouthing: Evidence from language production in German Sign Language,” in *The hands are the head of the mouth: The mouth as articulator in sign languag*, eds Boyes-Braem P. Sutton-Spence R. (Hamburg: Signum Press), 153–189.

[B30] Huguet E. (2016). *Les labialisations en LS: quels usages ont les récepeurs sourds des labialisations des mots français utilisées par les interprètes ?* [Master 2 Interprète LSF / Français. ].^****^city name.

[B31] Johnston T. (2012). Lexical Frequency in Sign Languages. *Journal of Deaf Studies and Deaf Education* 17 163–193. 10.1093/deafed/enr036 21841168

[B32] Johnston T. van Roekel J. Schembri A. (2016). On the Conventionalization of Mouth Actions in Australian Sign Language. *Language and Speech* 59 3–42. 10.1177/0023830915569334 27089804

[B33] Kimmelman V. (2009). Parts of speech in Russian Sign Language: The role of iconicity and economy. *Sign Language & Linguistics* 12 161–186. 10.1075/sll.12.2.03kim 33486653

[B34] Kubus O. Villwock A. Morford J. P. Rathmann C. (2015). Word recognition in deaf readers: Cross-language activation of German Sign Language and German. *Applied Psycholinguistics* 36 831–854. 10.1017/S0142716413000520

[B35] MacSweeney M. Waters D. Brammer M. J. Woll B. Goswami U. (2008). Phonological processing in deaf signers and the impact of age of first language acquisition. *NeuroImage* 40 1369–1379. 10.1016/j.neuroimage.2007.12.047 18282770 PMC2278232

[B36] Marinis T. (2010). “On-line sentence processing methods in typical and atypical populations,” in *Experimental Methods in Language Acquisition Research*, eds Unsworth S. Blom E. (Amsterdam: John Benjamins), 139–162.

[B37] Marshall J. Atkinson J. Woll B. Thacker A. (2005). Aphasia in a bilingual user of British signlanguage and english: Effects of cross-linguistic cues. *Cognitive Neuropsychology* 22 719–736. 10.1080/02643290442000266 21038274

[B38] Mayberry R. I. Witcher P. (2005). “Age of acquisition effects on lexical access in ASL: Evidence for the psychological reality of phonological processing in sign language,” in *Proceedings of the 30th Boston University conference on language development, Boston*.

[B39] McKee R. (2007). Hand to mouth: The role of mouthing in NSZL. *Across the Board: Australian Association of Sign Language Interpreters (Victoria)* 2 3–12.

[B40] Muir L. Richardson I. Leaper S. (2003). “Gaze tracking and its application to video coding,” in *Proceedings of the International Picture Coding Symposium*, Saint Malo.

[B41] Nadolske M. A. Rosenstock R. (2007). “Occurrence of mouthings in American Sign Language: A preliminary study,” in *Visible variation: Comparative studies in sign language structure*, eds Perniss P. Pfau R. Steinbach M. (Berlin: Mouton de Gruyter), 35–62.

[B42] Padden C. (1980). *Complement structures in American Sign Language*. San Diego: University of California.

[B43] Penner M. (2013). *The mouthing of verbs in Japanese Sign Language.* Ph.D. thesis. Grand Forks: University of North Dakota.

[B44] Perin P. Isel F. Bogliotti C. (0000). *Lexical database in French Sign Language*..^*****^(in progress). 10.3758/s13428-024-02521-1 39623247

[B45] Schermer T. (1985). “Analysis of natural discourse of deaf adults in the Netherlands: Observations on Dutch Sign Language,” in *Proceedings of the III International Symposium on Sign Language Research*, eds Stokoe W. C. Volterra V. (Silver Spring, MD: Linstok Press), 281–288.

[B46] Segui J. (2015). Évolution du concept de lexique mental. *Revue de Neuropsychologie et Neurosciences Cognitives et Cliniques* 7 1–6. 10.1684/nrp.2015.0325

[B47] Snodgrass J. G. Vanderwart M. (1980). A standardized set of 260 pictures: Norms for name agreement, image agreement, familiarity, and visual complexity. *Journal of Experimental Psychology: Human Learning and Memory* 6 174–215. 10.1037/0278-7393.6.2.174 7373248

[B48] Sutton-Spence R. (1999). The influence of English on British Sign Language. *International Journal of Bilingualism* 3 363–394. 10.1177/13670069990030040401

[B49] Sutton-Spence R. Day L. C. (2001). “Mouthings and Mouth Gestures in British Sign Language,” in *The Hands are the Head of the Mouth: The Mouth as Articulator in Sign Languages*, eds Boyes-Braem P. Sutton-Spence R. (Hamburg: Signum Press), (69–87).

[B50] Treisman A. M. (1960). Contextual cues in selective listening. *Quarterly Journal of Experimental Psychology* 12 242–248. 10.1080/17470216008416732

[B51] Vinson D. Thompson R. L. Skinner R. Fox N. Vigliocco G. (2010). The Hands and Mouth Do Not Always Slip Together in British Sign Language: Dissociating Articulatory Channels in the Lexicon. *Psychological Science* 21 1158–1167. 10.1177/0956797610377340 20644107

[B52] Vinson D. P. Cormier K. Denmark T. Schembri A. Vigliocco G. (2008). The British Sign Language (BSL) norms for age of acquisition, familiarity, and iconicity. *Behavior Research Methods* 40 1079–1087. 10.3758/BRM.40.4.1079 19001399

[B53] Weisenberg J. (2003). *Simultaneous codemixing in American Sign Language interpretation.* Ph.D. thesis. Stony Brook: State University of New York.

[B54] Woll B. (2001). “The sign that dares to speak its name: echo phonology in British Sign Language (BSL),” in *The Hands are the Head of the Mouth. The Mouth as Articulator in Sign Languages*, eds Boyes Braem P. Sutton-Spence R. (Hamburg, Germany: Signum Press), 87–98.

[B55] Woll B. (2014). Moving from hand to mouth: Echo phonology and the origins of language. *Frontiers in Psychology* 5:662. 10.3389/fpsyg.2014.00662 25071636 PMC4081976

[B56] Woll B. Mesch J. (2008). Frequency distribution and spreading behavior of different types of mouth actions in three sign languages. *Sign Lang. Linguistics* 11 45–67. 10.1075/sll.11.1.04cra 33486653

[B57] Woll B. Sieratzki J. S. (1998). Echo phonology: Signs of a link between gesture and speech. *Behavioral and Brain Sciences* 21 531–532. 10.1017/S0140525X98481263

